# Medical Journalism

**Published:** 1893-08

**Authors:** 


### *EDITORIAL.*

The office of THE JOURNAL is in room 330 Equitable Building.

Address communications relating to the Editorial Department to DR. L. B. GRANDY, Atlanta, Ga., Box 431.

Business communications should be addressed to DR. M. B. HUTCHINS, Atlanta, Ga., Box 431.

All remittances should be made payable to "ATLANTA MEDICAL AND SURGICAL JOURNAL."

Articles for publication should reach this office not later than the 15th instant.

The editors are not responsible for views expressed by contributors.

### MEDICAL JOURNALISM.

At the late meeting of the American Medical Editors' Association in Milwaukee, Mr. Ernest Hart, editor of the *British Medical Journal,* delivered a thoughtful and scholarly address on *Medical Journalism.* Mr. Hart spoke from an experience of thirty years in journalism. He regards medical journalism as a mission and no longer an "accidental function of an otherwise busy man's life." Those who have entered it in this spirit have failed to make much of a mark. Properly conducted it is a career that may "fulfil high ambitions and subserve large usefulness." But it is also a career of much sacrifice. Any sort of editorial work makes many enemies. The editor's work consists of literary fragments only; he never leaves one great monument of his genius.

Mr. Hart discussed the proper organization of a medical journal, and described the system by which he had conducted the *Journal of the British Medical Association* for many years. In that journal all editorial paragraphs are written by experts specially qualified for the subject discussed. For this purpose a staff of over two hundred writers is employed. Medical journals should be connected with some medical society, local or general.

We fear that the conditions under which Mr. Hart's own journal is conducted are very different from those which obtain in America. Those are the ideal conditions and would doubtless succeed anywhere in making an ideal journal. In our country the medical journal is often the property of some medical publisher, often that of the editors themselves, and too often the advertisement of some enterprising drug-house.

There are now about one hundred and eighty medical journals in the United States, and every few month witnesses the birth of more. Happily, also, every few months witnesses the death of others. Necessarily the inauguration of a new journal is an uncertain venture, and he who attempts it must have faith, and patience, and enterprise and perseverance. He must also have money, and be prepared to lose it. Since our own connection with this journal we have been interested in the new ones that have come to this office from time to time within the past two years. Some were born with a splutter and died exhausted. The minority have lived and are apparently healthy.

The organization of a new journal is attended with so many risks that, unless it is supplied with a capital which will run it until it gets good standing among physicians and advertisers, it must almost necessarily fail. Advertisers and physicians are slow to patronize any publication which has not the guarantee of continuance; and very properly so. The publication that has lived the longest in all probability will live the longest, and will receive the confidence of an increasing list of patrons.

We speak of this at this time because we hear of new journals forming, and because we have also heard within the last two or three months of the sad dissolution of two or three others. It does seem that in the medical world the tendency is to excess. There are too many medical journals, too many medical books, too many medical colleges; too many medicines, for that matter, and too many doctors, too. There are more medical journals now than are necessary to publish what is contributed to the medical press by the American profession. We do not need more journals, but we do need more of the spirit on the part of the profession to support by their subscriptions and encourage by their communications those that do exist and have shown themselves worthy of confidence. In this way a current medical literature could be built up of which we could all be proud, just as the English have reason to be proud of their few and high-class medical periodicals. Doubtless those who organize new journals do so from the laudable ambition to elevate medical journalism. We would rejoice at anything that would accomplish this. There are methods and there are methods, but the multiplication of journals is not one of them.

